# *Stat4* rs7574865 polymorphism promotes the occurrence and progression of hepatocellular carcinoma via the Stat4/CYP2E1/FGL2 pathway

**DOI:** 10.1038/s41419-022-04584-4

**Published:** 2022-02-08

**Authors:** Caie Wang, Na Gao, Lukui Yang, Yuanyuan Guo, Yan Fang, Tong Wang, Chen Xu, Gui fang Li, Jun Zhou, Yunfei Zhang, Qiang Wen, Hailing Qiao

**Affiliations:** 1 Institute of Clinical Pharmacology, School of Medicine, Zhengzhou University, Zhengzhou, Henan China; 2 Department of Pharmacy, the First Affiliated Hospital of Henan University of Science and Technology, Clinical Medical College of Henan University of Science and Technology, Luoyang, Henan China

**Keywords:** Cancer epigenetics, Cancer prevention, Oncogenes, Tumour-suppressor proteins

## Abstract

Although there are many studies on the relationship between genetic polymorphisms and the incidence of diseases, mechanisms are rarely known. We report the mechanism by which signal transducer and activator of transcription 4 (*stat4*) rs7574865 promotes the occurrence and progression of hepatocellular carcinoma (HCC). We found that the *GG* genotype at *stat4* rs7574865 was a risk genotype, and STAT4 levels in serum and peritumoral tissue from HCC patients with the *GG* genotype were significantly higher than those found in *TT* or *TG* carriers. Furthermore, HCC patients with the *GG* genotype or elevated STAT4 levels had poor prognoses. In vitro experiments demonstrated that STAT4 silencing promoted apoptosis and inhibited the invasion and migration of HepG2 and L02 cells. Proteomic analysis of HCC peritumors identified 273 proteins related to STAT4, of which CYP2E1 activity and FGL2 content exhibited the highest positive correlation. The relationship between CYP2E1 and FGL2 was also confirmed in *cyp2e1*^−/−^ mice and in CYP2E1 inhibitor-treated mice. In conclusion, this study elucidates the mechanism by which the *stat4* rs7574865 polymorphism promotes the occurrence and progression of HCC via the Stat4/CYP2E1/FGL2 pathway.

## Introduction

Hepatocellular carcinoma (HCC) is the most frequent form of primary liver cancer and is associated with a high mortality rate and poor progression. Recently, HCC was reported to be the fourth leading cause of cancer death worldwide. The development of HCC is a complex process that involves multiple risk factors, such as high HBV DNA levels, obesity, a family history of HCC, and alcohol consumption [1, 2]. Multiple reviews have shown that a family history of HCC is associated with a higher risk of HCC development and there are striking racial/ethnic disparities in HCC rates [1, 3]. Numerous studies have focused on the effects of genetic polymorphisms on HCC, such as the *HLA-DQ* gene [4–6]. It is worth noting that although there are many studies on the relationships between the incidence of diseases and genetic polymorphisms, the mechanisms by which genetic polymorphisms translate into disease are poorly understood.

The mechanisms arising from genetic polymorphisms may be complicated and involved in molecular networks. Therefore, it is difficult to elucidate the mechanisms by the study of a single or several proteins. Proteomics is a powerful tool for obtaining information on a great number of proteins simultaneously, including their expression levels and interactions with other molecules under specific conditions or circumstances [7, 8]. The application and development of proteomics is a novel approach to studying the mechanisms underlying genetic polymorphisms.

Signal transducers and activators of transcription (STATs) participate in the signal transduction of many cytokines [9, 10]. Seven mammalian STAT genes, including STAT1, 2, 3, 4, 5a, 5b, and 6, have been identified [11]. STAT4 is an important member of STATs and can be activated by interleukin (IL)-12. STAT4 is crucial for the differentiation of T helper (Th1) cells in promoting the cellular-mediated immune response [12] and has an important role in liver disease [13]. *Stat4* rs7574865 is related to autoimmune diseases such as multiple sclerosis, systemic lupus erythematosus (SLE), rheumatoid arthritis (RA), and primary Sjogren’s syndrome [14–17]. Recently, several studies have focused on the association between *stat4* rs7574865 and the risk of HCC, but the results are not consistent. A genome-wide association study (GWAS) conducted by Jiang found that a mutation (allele G) of *stat4* rs7574865 can significantly increase the risk of HCC and is associated with lower mRNA levels of STAT4 in HCC tissues [4]. However, the results of two other studies failed to find a relationship between this locus and HCC [18, 19]. Although there are reports on the effects of *stat4* rs7574865 on the occurrence of HCC, no study has disclosed the relevant mechanism, including the effects on progression.

In this study, the polymorphism and protein levels of STAT4 were determined in HCC patients. Proteomics technology was used to explore the molecular mechanism underlying the effect of the *stat4* rs7574865 genetic polymorphism on the occurrence and progression of HCC and could be of benefit in clinical practice.

## Materials and methods

### Human blood and liver sample collection

Blood samples were collected from 500 healthy people(Control) and 500 HCC patients at the First Affiliated Hospital of Zhengzhou University between the years 2013 and 2016. The inclusion criteria for all patients were: (1) HCC diagnosis as confirmed by experienced pathologists based on histological examination of HE-stained biopsy sections, B-ultrasound, CT, and MRI; (2) no anticancer treatment before surgery; (3) surgical resection, defined as the macroscopically complete removal of the tumor, with a histological demonstration of tumor-free margins; (4) available complete clinicopathologic and follow-up data. Serum was separated by centrifugation and stored at −20 °C, avoiding repeated freeze–thaw cycles prior to analysis.

In addition, a total of 42 healthy liver specimens and 42 HCC patient liver fibrosis tissue specimens were collected from Henan Provincial People’s Hospital and Affiliated Cancer Hospital of Zhengzhou University [20]. This study was approved by the Medical Ethics Committee of Zhengzhou University, and an informed consent form was obtained from all subjects. Liver tissue samples were snap-frozen in liquid nitrogen.

### Patient follow-up

A total of 314 patients with HCC who were diagnosed at the First Affiliated Hospital of Zhengzhou University were included for the survival analysis. Overall survival (OS) time of patients were followed up by telephone and outpatients were conducted after postoperation patients every 6 months, and for 42∼56 months until death or the last follow-up (last follow-up: December 2017). The maximum follow-up time (MFT) for the 314 patients was 2190 days and the median survival time (MST) was 1576 days. The follow-up by individuals who smoked one or more cigarettes per day for over 1 year were defined as smokers (ever-smokers were included) and those who consumed one or more alcoholic drinks a week for over 6 months were defined as alcohol drinkers (ever-drinkers were included). The median was used as a cutoff value to divide the patients into high or low expression groups.

### SNP genotyping

Genomic DNA was extracted from a leukocyte pellet by traditional proteinase K digestion, phenol–chloroform extraction, and ethanol precipitation. The SNP, rs7574865 T/G was genotyped using the 192.24 IFC Fluidigm SNP Type Assays (Applied Biosystems). The primers and probes for rs7574865 were as follows. Primers: sense, 50-TTTCCAGCACTTAATGAAAACACATAG, antisense, 50-CAAAGTTAAATTTCCCTGCTTTGAA; probes: allele T, FAM-TCTATGAGTCCGTATTGAGT-MGB, allele G, HEX-CTCTATGAGTCCATATTGAGT-MGB.

### STAT4 expression in serum and liver

The expression of STAT4 in different genotypes in human sera and liver tissues was detected with matched ELISA kits (DLSci and Tech Development, Wuxi) according to the manufacturer’s instructions. Samples were analyzed immediately at 450 nm on the full wavelength microplate reader (Thermo Fisher Technologies Co., LTD. USA).

### Cell culture and plasmid siRNA

Liver cancer cells HepG2 and Normal liver epithelial cells L02 were obtained from the Institute of Chemistry and Cell Biology (Shanghai, China). Both cells were free of mycoplasma contamination. Cells were cultured in Dulbecco’s modified Eagle’s medium (DMEM, Invitrogen, Carlsbad, CA, USA) supplemented with 10% fetal bovine serum (FBS) (HyClone, Logan, UT, USA) in an incubator at 37 °C, 5% CO_2_ and 100% humidity.

Silencer siRNA was constructed (Genechem, Shanghai, China). The STAT4 siRNA pool contains three siRNAs: siRNA-2180 (siRNA STAT4-1), siRNA-1776 (siRNA STAT4-2), and siRNA-1716 (siRNA STAT4-3). HepG2 and L02 cells were seeded in six-well culture plates with no antibiotic culture medium, and incubated at 37 °C for 24 h. The plasmid and Lipofectamine TM 3000 were diluted with serum-free DMEM before transfection, proportionally mixed and incubated for the fixed procedure. After 48 h, transfection efficiency was observed by fluorescence microscopy.

### Quantitative real-time PCR

Primers specific for STAT4 were obtained from Sango Biotech (Shanghai, China). Real-time PCR was performed using an ABI 7500 thermocycler with STAT4 or CYP2E1 primers and DNA templates and ROX Reference Dye (Takara, Japan). Values represent the average of three independent experiments. GAPDH was used as an internal control.

### Cell proliferation assay

The 3-(4, 5-dimethylthiazol-2-yl)-2, 5-diphenyltetrazolium bromide (MTT, Sigma, USA) assay was performed to evaluate the effect of STAT4 RNA interference on HepG2 and L02 cells proliferation. Absorbance was measured at 490 nm using a 2100 C ELISA Reader (Rayto Life and Analytical Sciences Co., Ltd, Shenzhen, China). The experiment was repeated three times. Proliferation inhibition rate = (blank control group absorbance − treated group absorbance)/(blank control group absorbance × 100%).

### Invasion/migration assays

Invasion and migration analysis were performed using a transwell chamber (Corning Corporation, USA). In the invasion assay, Matrigel (BD Biosciences, Bedford, MA, USA) was applied to the upper chamber. After transfection with siRNA-STAT4 or siRNA negative control for 48 h, HepG2 and L02 cells were reseeded onto the upper chambers of 24-well invasion inserts with 8 µm pore membranes in serum-free DMEM at a density of 3 × 10^5^ cells/well. The lower chamber was filled with a culture medium with 20% FBS and the cells were incubated for another 24 h. After incubation, the cells on the upper surface of the membrane were removed with cotton swabs, and cells invading across the Matrigel to the lower surface of the membrane were fixed with methanol and stained with crystal violet. Images were obtained under a microscope (Nikon, Chiyoda-Ku, Tokyo, Japan) at magnification (200×). The invaded cells were quantified by manual counting. Migration assays were carried out with a similar procedure, except that the polycarbonate membrane was not coated with Matrigel. Each experiment was repeated three times independently.

### Flow cytometry

HepG2 and L02 cells in the logarithmic growth phase were seeded in 6-well plates at 1 × 10^6^/well and cultured for 12 h. Plasmid transfection was performed when cells reached ~70% confluence. Apoptotic cells were analyzed by using Annexin V-APC/7-ADD Apoptosis Detection Kit (Nanjing KeyGen Biotech, Co. Ltd Jiangsu, China) according to the manufacturer’s protocol on Gallios flow cytometry (Beckman, CA, USA). The results were presented as the percentages of apoptotic cells relative to the total number of cells. The cell cycle was measured 48 h after transfection with a Cell Cycle Staining Kit (Multi Sciences, Hangzhou, China) according to the manufacturer’s protocol.

### Dual-luciferase reporter assay

The CYP2E1 promoter (forward: 5′-TTTCTCTATCGATAGGTACCCACGGGCAGAGGCACAGGCACTC-3′ and reverse:5′-CTTAGATCGCAGATCTCGAGGGTG CCGCTGGGGCCCTGCTGC-3′) was cloned into the pGL3-Basic vector (Promega, USA) to form a recombinant vector through restriction sites KpnI and XbaI, and was cotransfected with a plasmid of overexpression–stat4 and a negative control (NC) into HEK293T cells with Renilla luciferase as internal interference. After 48 h of transfection, the relative light unit (RLU) was measured by a Renilla luciferase assay kit and dual-luciferase reporter gene assay (Promega, USA). With Renilla luciferase as the internal reference, the dual-luciferase reporter gene assay system was employed for analysis. The activity of the target reporter gene was considered as the ratio of RLUs of firefly luciferase to that of Renilla luciferase. Each assay was performed in triplicate and repeated three times independently.

### Determination of CYP2E1 activity

Both human liver microsomes (HLMs) and mice liver microsomes (MLMs) were prepared by low-temperature differential centrifugation and the protein concentration was calculated using BCA protein quantification. A reaction system consisting of different concentrations of chlorzoxazone (CZX), liver microsomal protein, phosphate buffer, MgCl_2_, and EDTA was prepared, which was described as previously [21]. The reaction was started with NADPH and stopped with ethyl acetate. HPLC-UV was used to determine the amount of 6-hydroxy-CZX metabolite produced from the CYP2E1 substrate chlorzoxazone, and the human enzyme kinetic parameters such as maximum reaction rate (*V*_max_) and clearance (Cl_int_) were calculated. Mice CYP2E1 activity was determined by measuring the formation of 6-hydroxy-CZX.

### Label-free proteomics methods

Tissue protein was extracted and digested to obtain a peptide solution by filter-aided sample preparation [22]; the components were eluted and collected by high-pH reversed-phase chromatography. Phospho-peptide enrichment and fraction was by using titanium dioxide (TiO_2_) beads with tandem fractionation (TAFT) as previously described [23], with liquid chromatography tandem mass spectrometry (MS) analysis of peptide mixture. Finally, Max Quant software was used to analyze the data. The values of intensity-based absolute protein quantification (iBAQ) indicated the protein expression. The 41 and 71 samples were randomly selected from 105 normal and 102 para-cancerous liver tissues for samples preparation and MS analyses. According to quality control standards of proteomic data, 34 normal and 42 para-cancerous liver tissues were selected for subsequent analyses. The data files of proteome are available via ProteomeXchange with identifier PXD023118 (accession number PXD023118).

### Animal study

To establish the liver orthotopic tumor xenograft model, male BALB/c mice (*N* = 34), aging 8-week-old and weighing 20–25 g, were commercially acquired from purchased from Beijing Vital River Laboratory Animal Technology Co., Ltd. (Beijing, China), No:1100111911049459. Mice were anesthetized with 300 mg/kg chloral hydrate by intraperitoneal injection and then placed in a supine position on the operating table. To expose the left lobe of the liver, a 1.5 cm longitudinal incision was made below the xiphoid process. Mice were randomly divided into sham group, model group, and clomethiazole (CMZ) treatment group. CMZ was purchased from TCI Development Co., Ltd (Shanghai, China). CMZ is a widely used CYP2E1 inhibitor [24]. Mice of the model group and CMZ treatment group were injected with an H22 cells suspension and the sham group was injected with an equal amount of sterile saline. H22 hepatoma cells 1.5 × 10^6^/mL, 10 μL, sham group (*N* = 10); model group (*N* = 12); CMZ treatment group (*N* = 12). The mice were kept in a Specific Pathogen Free (SPF) warm incubator until they had recovered from the anesthesia and then returned to the animal room. After 3 weeks, the mice were killed and measured, and the liver samples were photographed, sectioned, and fixed in 10% formalin. The remaining portions of the liver were instantly frozen and stored in liquid nitrogen until used. After stripping the tumor, the tumor growth inhibition rate (incidence)was calculated.

Female and male C57BL/6 mice with a *cyp2e1* gene knockout, aging 8-week-old and weighing 20–25 g, were purchased from Beijing Bioset Gene Biotechnology Co., Ltd., license number: SCXK (Beijing) 2019-0001. Mice were raised in SPF environment. Offspring male mice were identified for *cyp2e1*^−/−^ mutant homozygous by PCR for subsequent experiments. To further explore whether CYP2E1 affects the expression of FGL2, the expression of FGL2 was measured in cyp2e1-null mouse liver tissue by western blot.

Mice were randomly captured and numbered successively, and grouped by SPSS Visual Binning according to the sample size of each group. All animal experiments were undertaken in accordance with the National Institute of Health Guide for the Care and Use of Laboratory Animals, with the approval of the Medical Ethics Committee of Zhengzhou University, Zhengzhou, China.

### Histology and immunohistochemistry

Formalin-fixed liver samples were embedded in paraffin, cut into 5-mm-thick sections, and stained with hematoxylin and eosin (HE) staining according to standard procedures.

### Western blot

Western blot was performed using standard procedures. Protein concentrations were determined with BCA protein assay kit (Beyotime Biotechnology, Shanghai, China). Dilution of all human antibodies, purchased from Abcam (Abcam, UK), are as follows: 1:500 anti-STAT4 antibody (ab284412), 1:300 anti-CYP2E1 antibody (ab28146), 1:2000 anti-GAPDH antibody (ab8245), 1:500 anti-FGL2 antibody (ab198029). Proteins were separated by 10% SDS-PAGE and transferred to a transfer membrane (Pall, USA) that was then incubated with an appropriate primary antibody. Anti-rabbit IgG conjugated to horseradish peroxidase was used as the secondary antibody (1:5000) for detection using an ECL kit (Kangwei Century Biological Technology Co., Ltd Beijing China), and band intensity was measured using Quantity One software version 4.6.2 (Bio-Rad Laboratories).

### Statistical analysis

Data were analyzed by SPSS version 22.0. Graphs were plotted by Graphpad Prism 8.3.0 software. The data were summarized as mean ± SD. Experimental results were analyzed by one-way analysis of variance (ANOVA) or Student’s *t*-test, with two-tailed *P*-value below 0.05 as a significant level. The binary logistic regression model was performed to determine differences in allele frequencies and genotype distributions between age- and sex-adjusted groups. Odds ratios (ORs) and their 95% confidence intervals (CIs) as well as the significance of deviations from Hardy–Weinberg equilibrium was assessed within patients by using goodness-of-fit *χ*^2^-test. Nonparametric tests were used for the analysis of normality test results. Receiver operating characteristics (ROC) curves were constructed to assess sensitivity, specificity, and respective areas under the curves (AUCs). The protein lowe or high expression group was divided by using median values (bottom).

## Results

### Influence of the *stat4* rs7574865 polymorphism on HCC

#### *Stat4 rs7574865* polymorphism and the occurrence of HCC

A total of 500 HCC patients were divided into two groups according to the *stat4 rs7574865* polymorphism: a group with the *TT* or *TG* genotypes and a group with *GG* genotype. The frequencies of genotypes and alleles of the *stat4* rs7574865 polymorphism are shown in Supplemental Table S1. Compared with the control group (46.12% and 68.21%, respectively), the frequency of both *GG* genotype and *G* allele were significantly increased (55.31% and 73.65%, respectively, *P* < 0.05). Odds ratio analysis showed that the *GG* genotype was associated with a significantly increased risk of HCC compared with the wild-type (OR = 1.45, 95% CI = 0.91–2.30). Our results suggest that the mutant homozygous *stat4* rs7574865 polymorphism may be a risk factor for the occurrence of HCC.

The basic characteristics and clinical parameters of studied subjects are in Supplementary Table S2. The allele frequency and genotype frequency distribution of *stat4* rs7574865 in the healthy control group and HCC were consistent with Hardy–Weinberg genetic balance law, indicating the genetic balance of the sample population in this study can represent the general population(Supplementary Table S3).

In addition, there was no significant difference in the distribution of heterozygous mutations between controls and HCC patients, so hereafter, this text combines *TT* and *TG* carriers into one group.

#### S*tat4 rs7574865* polymorphism and progression of HCC

**Fig. 1:**
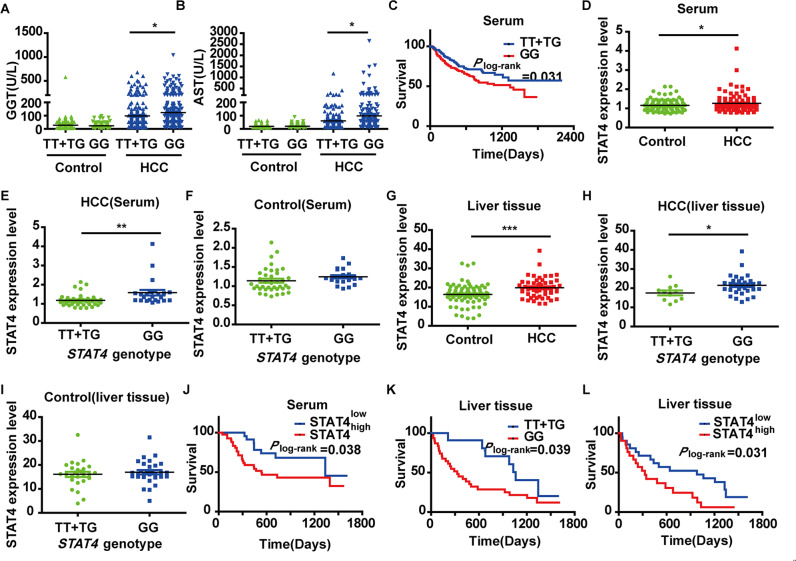
Association of the *stat4* rs7574865 genetic polymorphism with the risk of HCC. **A**, **B** The relationship between the *stat4* genetic polymorphism and clinical parameters: GGT (Control: *TT* + *TG* *N* = 127, *GG* *N* = 159; HCC: *TT* + *TG* *N* = 230, *GG* *N* = 269) and AST (Control: *TT* + *TG* *N* = 127, *GG* *N* = 159; HCC: *TT* + *TG* *N* = 230, *GG* *N* = 269) (*P < 0.05*, P-value* was calculated by Mann–Whitney *U*-test). **C** Kaplan–Meier survival curves for patients with HCC by *stat4* genetic polymorphism (Serum: *TT* + *TG* *N* = 136, *GG* *N* = 178) *P*-values are depicted by Cox–Mantel log-rank test. **D** STAT4 expression in HCC patients and controls in human sera (Control: *N* = 60; HCC: *N* = 65). **E**, **F** STAT4 expression with different genotypes in human serum (HCC: *TT* + *TG* *N* = 42, *GG* *N* = 23 and Control: *TT* + *TG* *N* = 38, *GG* *N* = 22). **G** STAT4 expression in HCC patients and controls in human liver tissue (Control: *N* = 56; HCC: *N* = 42). **H**, **I** STAT4 expression with different genotypes in human liver tissue (HCC: *TT* + *TG* *N* = 13, *GG* *N* = 31; Control: *TT* + *TG* *N* = 25, *GG* *N* = 31) *P < 0.05, *P < 0.01, and ***P < 0.001 was assessed by Student’s *t*-test. **J**, **L** Survival curves of HCC patients with high and low STAT4 expression (Serum: *N* = 65; liver tissue: *N* = 42). **K** Kaplan–Meier survival curves for patients with HCC by *stat4* genetic polymorphism (liver tissue: *TT* + *TG* *N* = 13, *GG* *N* = 31). *P*-values are depicted by Cox–Mantel log*-*rank test.

The results showed that GGT and AST levels were significantly higher in the HCC patients with the *GG* genotype (Fig. 1A, B). Kaplan–Meier survival analysis showed that the median survival time (MST) of patients with the *GG* genotype was 1338 days, while patients with the *TT* or *TG* genotype had not yet reached the MST until the last follow-up time of this study, suggesting that patients with the homozygous mutation (*GG*) experienced poor overall survival (Fig. 1C). Univariate analysis indicated that abnormal ALT, AST, GGT, GLB, PT, D-DT, FIB, DBIL, INR, CA125, CA199, and AFP was a higher risk of death when all other factors were cut off according to the abnormal clinical indicators except for age, gender, alcohol and tobacco history (Supplementary Table S4). Multivariate analysis further indicated that the *stat4 rs7574865* polymorphism was an independent risk factor for overall survival in HCC patients (Supplementary Table S5).

#### *rs7574865* polymorphism and the content of STAT4 in HCC patients

The STAT4 content in sera of 65 HCC patients was elevated compared with that in the 60 controls (*P* < 0.05, Fig. 1D). Interestingly, 23 *GG* genotype carriers had higher expression levels than 19 *TG* and 23 *TT* genotype carriers (*P* < 0.05, Fig. 1E) in patients with HCC, while the polymorphism had no effect on STAT4 content in the control group (*P* > 0.05, Fig. 1F). Furthermore, the STAT4 content in livers in another HCC patients and control was measured and the results were consistent with those measured in sera (*P* < 0.05, Fig. 1G–I). Our results suggest that genetic polymorphism of *stat4* might only affect the content of STAT4 in HCC patients, but does not affect the level of STAT4 in healthy people.

In addition, these patients were divided into two groups based on the content of STAT4 in sera. The results showed that the MST of the patients with STAT4^high^ group (535 days) was significantly decreased compared with the MST of patients with STAT4^low^ group (1335 days, *P* < 0.05, Fig. 1J), suggesting that HCC patients in the STAT4^high^ group have a poor progression.

For the 42 patients from which we collected liver specimens, the results were similar to the results obtained from the 500 HCC patients (Fig. 1K, L).

The above results suggest that the *rs7574865* polymorphism of *stat4* affects its expression in serum and liver tissue, and both the polymorphism and content affect the occurrence and prognosis of HCC. We speculate that the impact of the *rs7574865* polymorphism on HCC may be mediated by elevated STAT4 expression.

### Influence of STAT4 expression on HepG2 and L02 cells

**Fig. 2:**
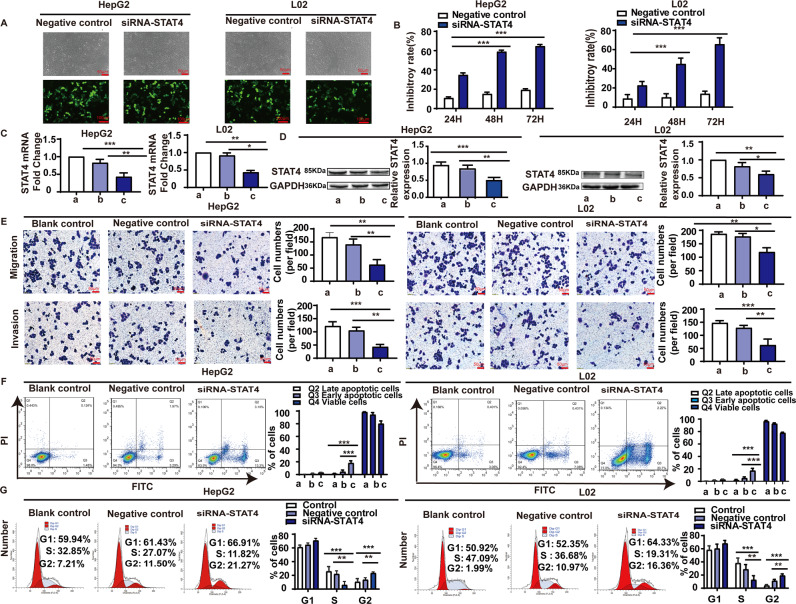
The effect of STAT4 on apoptosis, growth cycle, migration, and invasion in HepG2 and L02 cells. **A** The transfection efficiency was determined under a fluorescent microscope 48 h after transfection. The fluorescent siRNA (green) is evident in HepG2 and L02 cells. **B** Inhibition of cell proliferation by MTT assay at different time in HepG2 and L02 cells. **C** mRNA expression of STAT4 in HepG2 and L02 cells estimated by qPCR. **D** Expression of STAT4 and GAPDH in protein extracts from cultured HepG2 and L02 cells after 48 h by Western blot. Quantification of STAT4 is shown in the right picture. Data are representative of three independent experiments. **E** STAT4 siRNA inhibited the migration and invasion of HepG2 and L02 cells lines. **F** STAT4 siRNA promoted apoptosis in HepG2 and L02 cells. **G** STAT4 siRNA affected the growth cycle in HepG2 and L02 cells. Scale bar: 50 µm, 100 µm, *N* = 3. *P < 0.05, **P < 0.01, and ***P < 0.001 was assessed by one-way ANOVA.

HepG2 and L02 cells were transfected with pSilencer3.0-STAT4/siRNA expression vectors and the transfection efficiency was >60% as observed by a fluorescent microscope 48 h after transfection (Fig. 2A). The results of MTT showed that STAT4 siRNA significantly attenuated HepG2 and L02 cell proliferation and the inhibitory rate at 48 h and 72 h were 59.65 and 70.12% in HepG2 cells, and 45.09 and 65.69% in L02 cells, respectively (Fig. 2B). Both the mRNA and protein levels of STAT4 were significantly downregulated in HepG2 and L02 cells after transfection with STAT4 siRNA (Fig. 2C, D).

Transwell assays were used to detect the effect of STAT4 on the migration and invasion of HepG2 and L02 cells. The migration and invasion inhibition rates of the STAT4-siRNA group were 62.50 and 64.65% in HepG2 cells, 39.11 and 57.69% in L02 cells respectively (Fig. 2E).

In the STAT4-siRNA group, typical apoptotic morphological changes were observed, which manifested mainly as nuclear shrinkage, unequal size, irregular shape, and dense and strong fluorophores. Compared with the negative control, after transfection with STAT4 siRNA for 48 h, the early and late apoptosis rates of the STAT4-siRNA group were significantly decreased in HepG2 and L02 cells (Fig. 2F).

To elucidate the potential mechanism underlying cell growth inhibition induced by STAT4 silencing, cell cycle distribution was evaluated using flow cytometry. As shown in Fig. 2G, Compared with the negative controls, STAT4 knockdown significantly increased the percentage of cells in G2/M phase in HepG2 and L02 cells. Meanwhile, the number of S-phase HepG2 cells treated with siRNA-STAT4 was also markedly decreased, suggesting that knockdown of STAT4 might induce cell G2/M phase arrest. The results indicate that G_2_/M phase cell content increased significantly, while the S-phase cell content decreased significantly in HepG2 and L02 cells after silencing STAT4.

These results suggest that siRNA STAT4 could inhibit the proliferation, invasion, and migration of HepG2 and L02 cells and promote apoptosis.

### The mechanism of STAT4 in HCC as determined by proteomic analysis

**Fig. 3:**
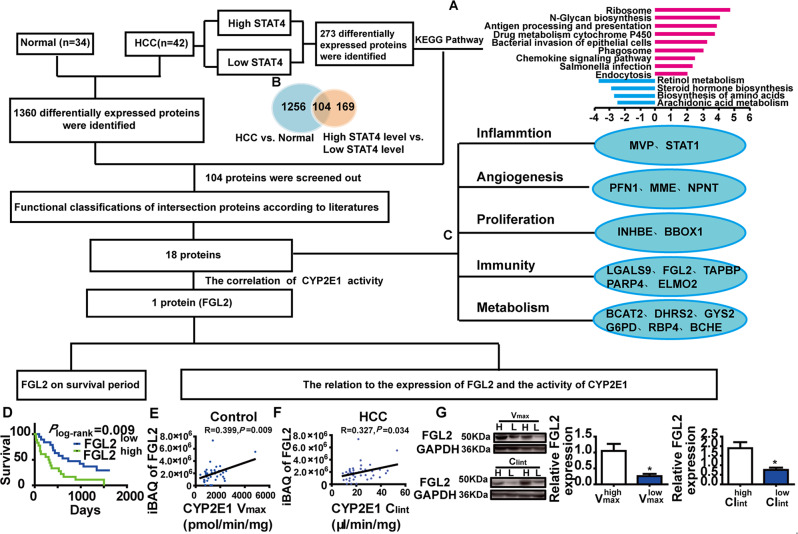
Schematic diagram to identify differentially expressed proteins resulting from the stat4 polymorphism: possible regulation of the CYP2E1/FGL2 pathway. **A** An enrichment analysis of the KEGG pathway for differentially expressed proteins in hepatofibrotic tissue of HCC patients. **B** Venn diagram showing the number of differential proteins in hepatofibrotic tissue of HCC patients. **C** Functional classification of differentially expressed proteins affected by the STAT4 expression and HCC: inflammation, angiogenesis, proliferation, immunity, and metabolism. Inflammation: MVP Major vault protein, STAT1 Signal transducer and activator of transcription 1. Angiogenesis: PFN Profilin-1, MME Neprilysin, NPNT Nephronectin. Proliferation: INHBE Inhibin beta E chain, BBOX1 Gamma-butyrobetaine hydroxylase1. Immunity: LGALS9 Galectin-9, FGL2 Fibroleukin (Fibrinogen-like protein 2), TAPBP Tapasin (TAP-binding protein); PARP4-Poly [ADP-ribose] polymerase 4; ELMO2 Engulfment and cell motility protein 2. Metabolism: BCAT2 Branched-chain amino acid aminotransferase, DHRS2 Dehydrogenase/reductase SDR family member 2; GYS2 Glycogen synthase, G6PD Glucose-6-phosphate 1-dehydrogenase, RBP4 Retinol binding protein 4, BCHE Choline-esterase. **D** Kaplan–Meier curve of overall survival for patients grouped by median of FGL2 expression levels, *P*-values are depicted by Cox–Mantel log-rank test. **E**, **F** The correlation between the expression of FGL2 and the activity of CYP2E1 (*V*_max_ and Cl_int_) in human HCC liver tissue, *R* and *P*-values by Pearson’s correlation test are depicted. **G** The expression of FGL2 on the median of *V*_max_ and Cl_int_ as the cutoff point, in HCC liver tissue by Western blot. GAPDH in each lane served as an internal control for normalization (*N* = 3), *P < 0.05 was assessed by Student’s *t*-test.

To analyze the molecular mechanisms by which the *stat4 rs7574865* polymorphism promotes HCC, fibrotic livers from 42 HCC patients were selected for proteomic analysis. According to the median expression level of STAT4 in the livers, HCC patients were divided into two groups: a STAT4^high^ group and a STAT4^low^ group. Comparison of these groups revealed 273 differentially expressed proteins. A pathway enrichment analysis by KEGG revealed upregulation of metabolism-related pathways, including CYP2E1 (Fig. 3A). Of these pathways, our previous data demonstrated that CYP2E1 metabolic activity was significantly increased in livers from HCC patients [21], and inhibition of CYP2E1 activity significantly decreased liver injury and liver carcinogenesis in rats induced by diethylnitrosamine (DEN) [25]. Therefore, we speculate that STAT4 effects on the occurrence and progression of HCC might be via regulation of CYP2E1.

In addition, 1360 differentially expressed proteins were found by comparison of HCC and control samples, and 104 proteins matched the 273 proteins identified above (Fig. 3B), suggesting that these 104 proteins are not only associated with HCC but also related to STAT4. A literature search confirmed that 18 of these 104 differentially expressed proteins were reported to be related to the occurrence and progression of HCC. Functional classification of these 18 proteins includes immunity, inflammation, angiogenesis, metabolism, and proliferation (Fig. 3C).

Among the 18 proteins, only FGL2 was not only highly expressed in HCC patients, but also related to CYP2E1 metabolic activity. The expression of FGL2 exhibited a high correlation with the activity of CYP2E1. Kaplan–Meier analysis revealed that the higher expression of FGL2 was significantly associated with shorter overall survival (Fig. 3D). Compared with patients with low *V*_max_ or Cl_int_, for CYP2E1, the iBAQ value of FGL2 was significantly higher than that in patients with high *V*_max_ or Cl_int_ (Fig. 3E, F). Furthermore, these results were confirmed by western blot (Fig. 3G). The results suggest that FGL2 might be downstream of CYP2E1 in the STAT4/CYP2E1 pathway and that STAT4 affects the occurrence and progression of HCC via regulation of the CYP2E1/FGL2 pathway.

### The regulation by STAT4 of CYP2E1

**Fig. 4:**
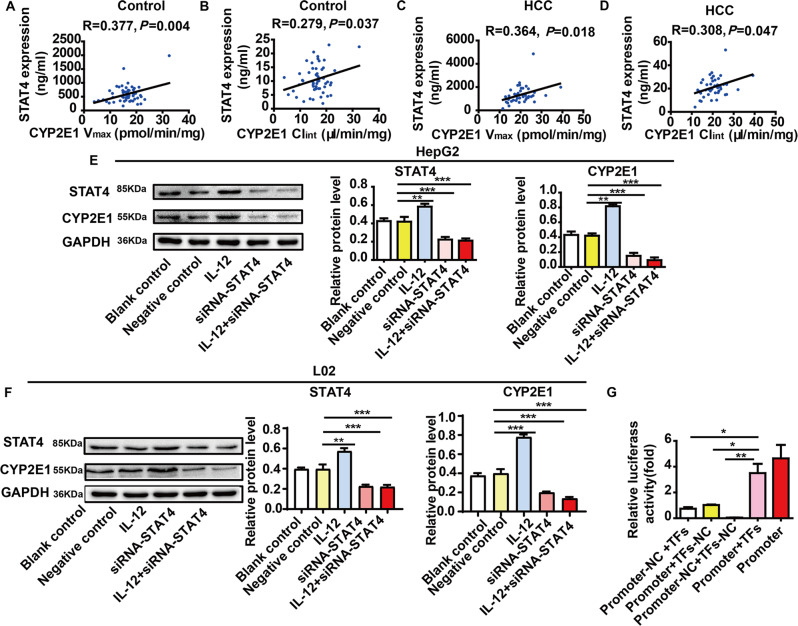
The regulation by STAT4 of CYP2E1. **A**–**D** The correlation between content of STAT4 and activity of CYP2E1 (*V*_max_ and Cl_int_) was analyzed. The result revealed that content of STAT4 was significantly positively correlated with the *V*_max_ and Cl_int_ in control and HCC liver tissues (*N* = 42, R and *P*-values by Pearson’s correlation test are depicted). **E**, **F** The expression of STAT4 and CYP2E1 in HepG2 and L02 cells in the IL-12 treated group and the STAT4 siRNA group by western blot (*N* = 3). GAPDH in each lane served as an internal control for normalization. HepG2 and L02 cells for 48 h with blank control group, negative control group, IL-12 group, and siRNA-STAT4 group. Data are representative of three independent experiments. **G** The effect of STAT4 on CYP2E1 promoter activity was detected by double luciferase reporter gene. Promoter-NC + TFs: STAT4 (TFs) and CYP2E1 promoter negative control plasmid; (CYP2E1 promoter + TFs-NC group): CYP2E1 promoter and STAT4-negative control plasmid (TFs-NC) group; CYP2E1 promoter-NC and TFs-NC plasmid group; CYP2E1 promoter and TFs (STAT4); Promoter: CYP2E1 promoter normal plasmid group (*N* = 3). *P < 0.05, **P < 0.01, and ***P < 0.001 was assessed by one-way ANOVA.

The correlation between the content of STAT4 and the activity of CYP2E1 (*V*_max_ and Cl_int_) was assessed by Spearman’s test. The results revealed that both *V*_max_ and Cl_int_ of CYP2E1 had positive correlation with STAT4 expression in 42 HCC livers and 34 normal livers (Fig. 4A–D).

To study the regulation of CYP2E1 by STAT4, the expression of STAT4 in HepG2 and L02 cells was silenced and induced by STAT4 siRNA and IL-12, respectively. The results showed that the protein levels of CYP2E1 were significantly decreased in HepG2 and L02 cells transfected with STAT4 siRNA and significantly increased when induced by IL-12 (Fig. 4E, F).

A double luciferase reporter gene was used to detect the effect of STAT4 on CYP2E1 promoter activity. HEK293T cells were transfected with a plasmid with a dual-luciferase expression system through the CYP2E1 promoter sequence (pGL3-basic) and transcription of STAT4 expression. The relative luciferase activity of the CYP2E1 promoter + TFs group was significantly higher than the relative luciferase activity of the CYP2E1 promoter-NC and TFs-NC plasmid group (Fig. 4G). For the CYP2E1 promoter-reporter gene, the luciferase labeled with STAT4 increased significantly, indicating that STAT4 regulates the CYP2E1 promoter region.

### The regulation by CYP2E1 of FGL2

#### The relationship between FGL2 and HCC

**Fig. 5:**
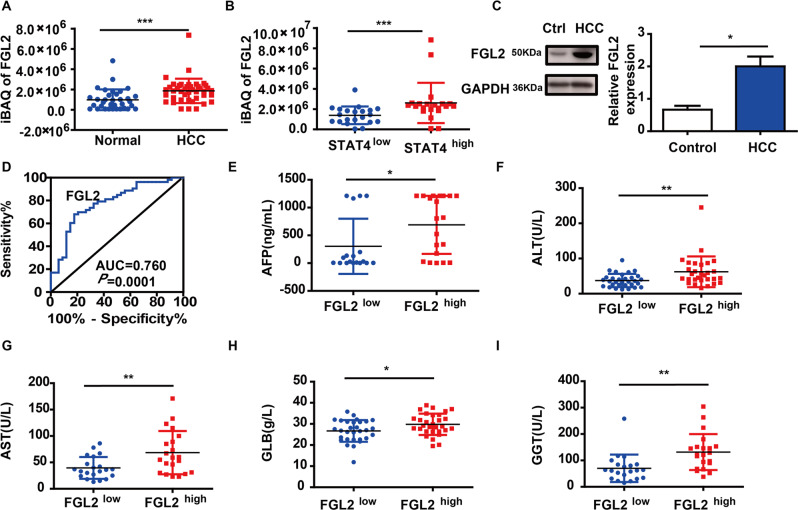
The relationship between FGL2 and HCC. **A** Comparison of FGL2 expression levels between the normal group (*N* = 34) and the HCC group (*N* = 42). **B** Comparison of FGL2 expression levels between the low STAT4 group (*N* = 21) and the high STAT4 group (*N* = 21). **C** The expression of FGL2 in liver tissue by western blot (*N* = 6), *P < 0.05, ***P < 0.001 was assessed by Student’s *t*-test. **D** ROC curve analysis for FGL2, AUC indicated area under ROC curve. **E**–**I** The relationship between the expression of FGL2 and clinical indicators (AFP, ALT, AST, GLB, GGT). *P < 0.05, **P < 0.01, and ***P < 0.001 was assessed by Student’s *t*-test.

The proteomics results showed that FGL2 expression in HCC patients was significantly higher than that of controls (*P* < 0.05, Fig. 5A). Moreover, the expression of FGL2 increased in the STAT4^high^ group compared with the STAT4^low^ group (*P* < 0.05, Fig. 5B). The result was also confirmed that FGL2 expression was significantly higher in HCC patients by western blot (Fig. 5C). Furthermore, the AUC of ROC curve of FGL2 was 0.760 (Fig. 5D).

Based on the median expression level of FGL2 in liver, HCC patients were classified into two groups: the FGL2^high^ group and the FGL2^low^ group. The results showed that several clinicopathological traits, including AFP, ALT, AST, GLB, and GGT were significantly higher in the FGL2^high^ group than that in FGL2^low^ group (*P* < 0.05, Fig. 5E–I).

#### The regulation by CYP2E1 of FGL2 expression

**Fig. 6:**
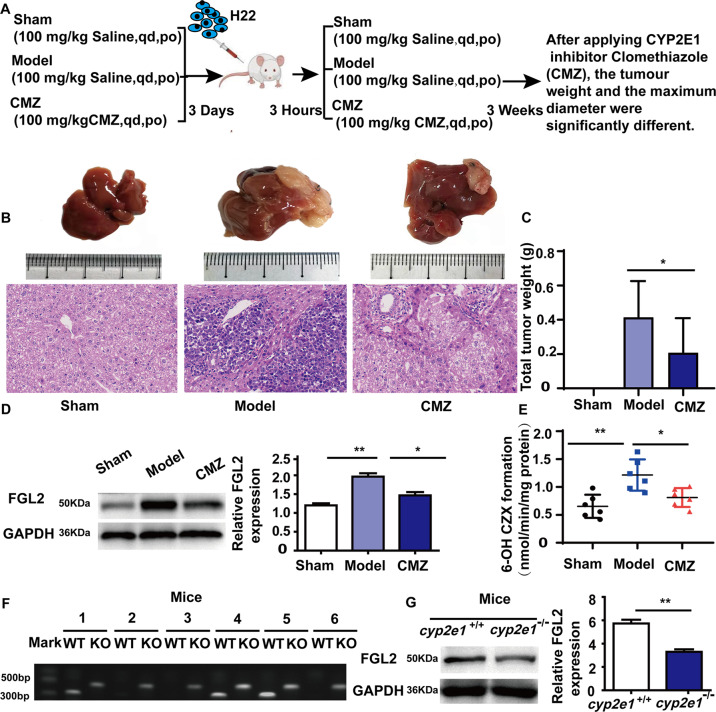
CYP2E1 regulates STAT4-mediated FGL2 expression. **A** Experimental scheme of the effect of CMZ on FGL2 in BALB/c mice. **B** Representative images of macroscopic liver (top) and H&E staining (bottom) (scale bar, 50 µm). **C** The tumor weight; sham group (*N* = 10), model group (*N* = 12), CMZ group (*N* = 12), *P < 0.05, **P < 0.01 was assessed by one-way ANOVA. **D** The expression of FGL2 in liver tissues in an orthotopic transplantation tumor model of H22 cells in BALB/c mice by western blot (*N* = 3). **E** The 6-hydroxy-CZX formation (*N* = 6), *P < 0.05 and **P < 0.01 was assessed by one-way ANOVA. **F** The effect of *cyp2e1* knockout in mice was identified by PCR. Mice no. 1, no. 2, no. 3, and no. 5 only showed bands around 520 bp, and no bands around 464 bp, which were mutant homozygous (*N* = 6). Mice no. 4 and no. 6 exhibited two bands near 464 bp and 520 bp, respectively, indicating that they were heterozygous. **G** The expression of FGL2 in liver tissues in (*N* = 3). **P < 0.01 was assessed by Student’s *t*-test.

In addition, the relationship between CYP2E1 and FGL2 was further confirmed in HCC mice treated with CMZ. A mouse orthotopic xenograft model with H22 cells was prepared, and the mice were randomly divided into sham group, model group, and CMZ group (Fig. 6A). The tumor incidence rates were 100% and 66.67% in liver tumor model and CMZ group, respectively. HE staining was used to assess tumor formation. Macroscopically, there were significant differences in tumor weight in the group treated with CMZ decreased remarkably (Fig. 6B). The tumor weight in the CMZ group was decreased in the model group (*P* < 0.05, Fig. 6C). The results also show that the expression of FGL2 in the model group was significantly increased, while FGL2 content decreased in the group treated with CMZ (*P* < 0.05, Fig. 6D), which suggested that the inhibitory effect of the CYP2E1 inhibitor on the liver cancer mouse model may be related to the decreased expression of FGL2. The 6-hydroxy-CZX formation in the model group was significantly increased than in the sham group and CMZ, which suggested that CYP2E1 activity might be decreased in the group treated with CMZ (*P* < 0.01, *P* < 0.05, Fig. 6E). To further explore the regulation of FGL2 by CYP2E1, a *cyp2e1* gene knockout (*cyp2e1*^−/−^) mouse was established (Fig. 6F). The results showed that the expression of FGL2 in *cyp2e1*^−/−^ mice was significantly decreased compared with the expression in wild-type mice (*P* < 0.05, Fig. 6G).

The above results indicate that the expression of FGL2 might be regulated by CYP2E1.

**Fig. 7:**
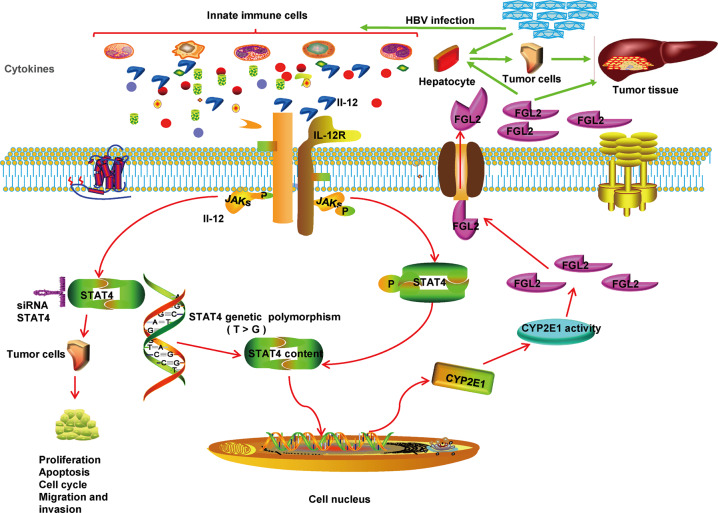
Graphical representation of the possible mechanism by which the *stat4* genetic polymorphism affects HCC. STAT4 has dual roles during HCC (i) STAT4 directly promotes HepG2 cell proliferation, migration, and invasion, and affects apoptosis and cell cycle; (ii) Polymorphism rs7574865 participates the occurrence and progression of HCC by regulating the CYP2E1/FGL2 pathway.

In conclusion, the novel findings of this study are summarized in Fig. 7. Polymorphism rs7574865 of *stat4* promotes the occurrence and progression of HCC by regulating the Stat4/CYP2E1/FGL2 pathway. Mutant homozygotes of the *stat4* rs7574865 polymorphism cause an increase in STAT4 expression, and STAT4 promotes the expression of CYP2E1 by regulating the promoter region of CYP2E1. Increased CYP2E1 activity upregulates the expression of FGL2 in HCC.

## Discussion

There have been some reports on the relationship between *stat4* rs7574865 and HCC, but none have identified the mechanism by which this polymorphism promotes HCC. We studied 500 control and 500 HCC patients and found that the *GG* genotype at *stat4* rs7574865 was a risk genotype for HCC, and the STAT4 protein content in serum and livers of *GG* carriers was significantly higher than the STAT4 content in the serum and livers of *TG* and *TT* carriers. Furthermore, HCC patients with the *GG* genotype or higher STAT4 expression have a poor progression. Proteomic analysis suggests that the underlying mechanism by which the *stat4* rs7574865 polymorphism promotes the occurrence and progression of HCC is via the Stat4/CYP2E1/FGL2 pathway.

As a member of the STAT family, STAT4 can regulate the expression of inflammatory mediators of T cells and NK cells and plays an important role in a variety of pathophysiological processes such as immunity and tumor growth [10, 12, 26, 27]. It was reported that STAT4 mRNA or protein was highly expressed in breast cancer [28], lung cancer [29], colorectal cancer [30]. Cheng et al. [30] found that *stat4* knockdown can inhibit the growth and invasion of colorectal cancer cells. This study also found that the protein of level of STAT4 in livers and serum from HCC patients was significantly higher than that of controls and that silencing STAT4 expression in HepG2 and L02 cells promoted cell apoptosis and inhibited cell migration and invasion. Several previous studies [4, 31, 32] found that the mRNA expression of STAT4 in HCC tumor tissues was significantly lower than that in adjacent nontumor liver tissues. However, the above study measured the mRNA level, while this study focused on the protein level of STAT4. Moreover, our results show the differences between fibrotic livers from HCC patients and normal livers.

At present, few studies on disease susceptibility genes have involved the underlying mechanism from gene to disease. The rapid development of proteomics has provided new approaches for studying the underlying mechanisms. This study found that mutation at *stat4* rs7574865 promoted the occurrence and progression of HCC by affecting the expression of STAT4. Our group firstly explored the related mechanisms by proteomics and found that metabolism-related pathways were upregulated, including CYP2E1. CYP2E1 is an important member of the cytochrome P450 enzyme family, which is involved not only in the metabolism of ~6% of drugs but also is involved in inflammation-related diseases [33, 34]. CYP2E1 plays a key role in the occurrence and progression of liver fibrosis and liver cancer [25, 35]. The results of Kang et al. [36] indicated that the occurrence of liver cancer in *cyp2e1* knockout mice induced by DEN was significantly decreased. Our previous studies found that CYP2E1 activity was higher in human fibrotic livers than that in normal livers [21], and higher CYP2E1 activity was correlated with hepatocarcinogenesis induced by DEN [25]. Recent studies have shown that STAT family members such as STAT1, STAT3, and STAT6 can regulate the transcription and posttranslational modification of CYP2E1 [37–39]. This study further demonstrates that STAT4 could regulate the expression of CYP2E1 by regulating the CYP2E1 promoter region.

Based on the expression level of STAT4 in livers, 273 differentially expressed proteins were identified by proteomics. Among these proteins, the expression of 104 proteins was significantly higher in HCC patients than in controls, of which 18 proteins previously had been reported to be related to the occurrence and progression of HCC. Of these, the expression of FGL2 was related to the activity of CYP2E1, so we speculated that CYP2E1 might regulate or be regulated by FGL2.

FGL2, also known as FGL2 prothrombinase, is a member of the fibrinogen-related protein superfamily [40]. FGL2 was reported to be not only significantly increased in hepatitis [41, 42] and nonalcoholic fatty liver [43] but also highly expressed in liver cancer [44, 45]. The results of Liu indicated that knocking out FGL2 expression can significantly inhibit the growth of liver implant tumors and slow down the progression of liver cancer [45]. In this study, we verified the relationship between CYP2E1 and FGL2 by using *cyp2e1* knockout mice and found that FGL2 levels decreased significantly when *cyp2e1* was deleted. Moreover, in the mouse models of liver cancer, CYP2E1 inhibitors not only reduced the incidence and size of tumors but also significantly decreased the expression of FGL2.

The mechanism by which CYP2E1 regulates FGL2 is not clear and should be further studied. CYP2E1 could promote hepatic oxidative stress by generating ROS [46]. In viral infectious hepatitis, excessive activation of the ROS/NLRP3/IL-1β axis resulted in aggravation of hepatitis. The main mechanism of action by which IL-1β was triggered by ROS combined with TNF-α to activate NF-κB to induce FGL2 expression, which intensifies the progression of hepatitis. The high expression of CYP2E1 was closely associated with the increasing release of multiple of proinflammatory cytokines [39]. It was also reported that a CYP2E1 inhibitor can significantly inhibit ethanol-induced overexpression of CYP2E1 in Kupffer cells, thereby repressing the increase in ROS production and the activation of NF-κB, and ultimately reducing the increase in TNF-α production [47]. In human retinal pigment epithelial cells, a CYP2E1 inhibitor, quercetin [48], can decrease the production of IL-1β by blocking the activation of MAPK and NF-κB signaling pathways [49]. In cardiac microvascular endothelial cells, inhibition of activation of NF-κB can suppress the upregulation of FGL2 induced by TNF-α [50]. Overall, we speculate that CYP2E1 may participate in the regulation of FGL2 expression through ROS or inflammatory cytokines triggered downstream.

In conclusion, our proteomically based results have innovatively revealed that the *stat4* rs7574865 polymorphism promotes the occurrence and progression of hepatocellular carcinoma via the Stat4/CYP2E1/FGL2 pathway. The results present a new theoretical basis for clarifying the mechanism of HCC occurrence and progression and reveal a new therapeutic target. Moreover, this paper provides new ideas for identifying the mechanisms by which gene mutations lead to disease.

## Supplementary information

Table S1

Table S2

Table S3

Table S4

Table S5

Related Manuscript File

Figure legends

Supplementary Table legends.

Highlights

Related Manuscript File

## Data Availability

The data that support the findings of this study are available from the corresponding author upon reasonable request. The data files of proteome are available via ProteomeXchange with identifier PXD023118 (accession number PXD023118).

## References

[1] McGlynn KA, Petrick JL, El-Serag HB (2021). Epidemiology of hepatocellular carcinoma. Hepatology..

[2] Bray F, Ferlay J, Soerjomataram I, Siegel RL, Torre LA, Jemal A (2018). Global cancer statistics 2018: GLOBOCAN estimates of incidence and mortality worldwide for 36 cancers in 185 countries. CA Cancer J Clin..

[3] Lyu X, Liu K, Chen Y, Wang Z, Yao J, Cai G (2016). Analysis of risk factors associated with the development of hepatocellular carcinoma in chronic HBV-infected Chinese: a meta-analysis. Int J Environ Res Public Health.

[4] Jiang DK, Sun J, Cao G, Liu Y, Lin D, Gao YZ (2013). Genetic variants in STAT4 and HLA-DQ genes confer risk of hepatitis B virus-related hepatocellular carcinoma. Nat Genet.

[5] Kumar V, Kato N, Urabe Y, Takahashi A, Muroyama R, Hosono N (2011). Genome-wide association study identifies a susceptibility locus for HCV-induced hepatocellular carcinoma. Nat Genet.

[6] Lee MH, Huang YH, Chen HY, Khor SS, Chang YH, Lin YJ (2018). Human leukocyte antigen variants and risk of hepatocellular carcinoma modified by hepatitis C virus genotypes: A genome-wide association study. Hepatology..

[7] Matsumoto M, Nakayama KI (2018). The promise of targeted proteomics for quantitative network biology. Curr Opin Biotechnol..

[8] McBride AA (2017). The promise of proteomics in the study of oncogenic viruses. Mol Cell Proteom..

[9] Darnell JE, Kerr IM, Stark GR (1994). Jak-STAT pathways and transcriptional activation in response to IFNs and other extracellular signaling proteins. Science..

[10] Iida K, Suzuki K, Yokota M, Nakagomi D, Wakashin H, Iwata A (2011). STAT4 is required for IFN-beta-induced MCP-1 mRNA expression in murine mast cells. Int Arch Allergy Immunol..

[11] Horvath CM (2000). STAT proteins and transcriptional responses to extracellular signals. Trends Biochem Sci..

[12] Glosson-Byers NL, Sehra S, Kaplan MH (2014). STAT4 is required for IL-23 responsiveness in Th17 memory cells and NKT cells. JAKSTAT..

[13] Wang Y, Qu A, Wang H (2015). Signal transducer and activator of transcription 4 in liver diseases. Int J Biol Sci..

[14] Nageeb RS, Omran AA, Nageeb GS, Yousef MA, Mohammad YAA, Fawzy A (2018). STAT4 gene polymorphism in two major autoimmune diseases (multiple sclerosis and juvenile onset systemic lupus erythematosus) and its relation to disease severity. Egypt J Neurol Psychiatr Neurosurg..

[15] Beltran Ramirez O, Mendoza Rincon JF, Barbosa Cobos RE, Aleman Avila I, Ramirez Bello J (2016). STAT4 confers risk for rheumatoid arthritis and systemic lupus erythematosus in Mexican patients. Immunol Lett..

[16] Gupta V, Kumar S, Pratap A, Singh R, Kumari R, Kumar S (2018). Association of ITGAM, TNFSF4, TNFAIP3 and STAT4 gene polymorphisms with risk of systemic lupus erythematosus in a North Indian population. Lupus..

[17] Zheng J, Yin J, Huang R, Petersen F, Yu X (2013). Meta-analysis reveals an association of STAT4 polymorphisms with systemic autoimmune disorders and anti-dsDNA antibody. Hum Immunol..

[18] Clark A, Gerlach F, Tong H, Hoan NX, Song le H, Toan NL (2013). A trivial role of STAT4 variant in chronic hepatitis B induced hepatocellular carcinoma. Infect Genet Evol..

[19] Chen K, Shi W, Xin Z, Wang H, Zhu X, Wu X (2013). Replication of genome wide association studies on hepatocellular carcinoma susceptibility loci in a Chinese population. PLoS One..

[20] Gao J, Wang Z, Wang GJ, Gao N, Li J, Zhang YF (2018). From hepatofibrosis to hepatocarcinogenesis: higher cytochrome P450 2E1 activity is a potential risk factor. Mol Carcinog..

[21] Zhou J, Wen Q, Li SF, Zhang YF, Gao N, Tian X (2016). Significant change of cytochrome P450s activities in patients with hepatocellular carcinoma. Oncotarget..

[22] Jiang Y, Sun A, Zhao Y, Ying W, Sun H, Yang X (2019). Proteomics identifies new therapeutic targets of early-stage hepatocellular carcinoma. Nature..

[23] Wisniewski JR, Zougman A, Nagaraj N, Mann M (2009). Universal sample preparation method for proteome analysis. Nat Methods..

[24] Stresser DM, Perloff ES, Mason AK, Blanchard AP, Dehal SS, Creegan TP (2016). Selective time- and NADPH-dependent inhibition of human CYP2E1 by Clomethiazole. Drug Metab Dispos..

[25] Gao J, Wang Z, Wang GJ, Zhang HX, Gao N, Wang J (2018). Higher CYP2E1 activity correlates with hepatocarcinogenesis induced by diethylnitrosamine. J Pharm Exp Ther..

[26] Watford WT, Hissong BD, Bream JH, Kanno Y, Muul L, O’Shea JJ (2004). Signaling by IL-12 and IL-23 and the immunoregulatory roles of STAT4. Immunol Rev..

[27] Yu S, Jia L, Zhang Y, Zhong J, Yang B, Wu C (2015). IL-12 induced the generation of IL-21- and IFN-gamma-co-expressing poly-functional CD4+ T cells from human naive CD4+ T cells. Cell Cycle..

[28] Zhou J, Xu XZ, Hu YR, Hu AR, Zhu CL, Gao GS (2014). Cryptotanshinone induces inhibition of breast tumor growth by cytotoxic CD4+ T cells through the JAK2/STAT4/ perforin pathway. Asian Pac J Cancer Prev..

[29] Yoon YH, Hwang HJ, Sung HJ, Heo SH, Kim DS, Hong SH (2019). Upregulation of complement factor H by SOCS-1/3(-)STAT4 in lung cancer. Cancers (Basel).

[30] Cheng JM, Yao MR, Zhu Q, Wu XY, Zhou J, Tan WL (2015). Silencing of stat4 gene inhibits cell proliferation and invasion of colorectal cancer cells. J Biol Regul Homeost Agents..

[31] Kim LH, Cheong HS, Namgoong S, Kim JO, Kim JH, Park BL (2015). Replication of genome wide association studies on hepatocellular carcinoma susceptibility loci of STAT4 and HLA-DQ in a Korean population. Infect Genet Evol..

[32] Wubetu GY, Utsunomiya T, Ishikawa D, Yamada S, Ikemoto T, Morine Y (2014). High STAT4 expression is a better prognostic indicator in patients with hepatocellular carcinoma after hepatectomy. Ann Surg Oncol..

[33] Abu-Serie MM, El-Gamal BA, El-Kersh MA, El-Saadani MA (2015). Investigation into the antioxidant role of arginine in the treatment and the protection for intralipid-induced non-alcoholic steatohepatitis. Lipids Health Dis..

[34] Seth RK, Das S, Pourhoseini S, Dattaroy D, Igwe S, Ray JB (2015). M1 polarization bias and subsequent nonalcoholic steatohepatitis progression is attenuated by nitric oxide donor DETA NONOate via inhibition of CYP2E1-induced oxidative stress in obese mice. J Pharm Exp Ther..

[35] Gao J, Wang GJ, Wang Z, Gao N, Li J, Zhang YF (2017). High CYP2E1 activity correlates with hepatofibrogenesis induced by nitrosamines. Oncotarget..

[36] Kang JS, Wanibuchi H, Morimura K, Gonzalez FJ, Fukushima S (2007). Role of CYP2E1 in diethylnitrosamine-induced hepatocarcinogenesis in vivo. Cancer Res..

[37] Wang J, Hu Y, Nekvindova J, Ingelman-Sundberg M, Neve EP (2010). IL-4-mediated transcriptional regulation of human CYP2E1 by two independent signaling pathways. Biochem Pharmacol..

[38] Pan J, Xiang Q, Ball S, Scatina J, Kao J, Hong JY (2003). Lipopolysaccharide-mediated modulation of cytochromes P450 in Stat1 null mice. Drug Metab Dispos..

[39] Patel SA, Bhambra U, Charalambous MP, David RM, Edwards RJ, Lightfoot T (2014). Interleukin-6 mediated upregulation of CYP1B1 and CYP2E1 in colorectal cancer involves DNA methylation, miR27b and STAT3. Br J Cancer.

[40] Yanaba K, Asano Y, Noda S, Akamata K, Aozasa N, Taniguchi T (2013). Increased circulating fibrinogen-like protein 2 in patients with systemic sclerosis. Clin Rheumatol..

[41] Foerster K, Helmy A, Zhu Y, Khattar R, Adeyi OA, Wong KM (2010). The novel immunoregulatory molecule FGL2: a potential biomarker for severity of chronic hepatitis C virus infection. J Hepatol..

[42] Marsden PA, Ning Q, Fung LS, Luo X, Chen Y, Mendicino M (2003). The Fgl2/fibroleukin prothrombinase contributes to immunologically mediated thrombosis in experimental and human viral hepatitis. J Clin Invest..

[43] Colak Y, Senates E, Ozturk O, Yilmaz Y, Coskunpinar E, Kahraman OT (2011). Plasma fibrinogen-like protein 2 levels in patients with non-alcoholic fatty liver disease. Hepatogastroenterology..

[44] Yang M, Zhang Z, Chen J, Xu M, Huang J, Wang M (2019). Soluble fibrinogen-like protein 2 promotes the growth of hepatocellular carcinoma via attenuating dendritic cell-mediated cytotoxic T cell activity. J Exp Clin Cancer Res..

[45] Liu Y, Xu L, Zeng Q, Wang J, Wang M, Xi D (2012). Downregulation of FGL2/prothrombinase delays HCCLM6 xenograft tumour growth and decreases tumour angiogenesis. Liver Int..

[46] Ma L, Wang Y, Chen X, Zhao L, Guo Y (2020). Involvement of CYP2E1-ROS-CD36/DGAT2 axis in the pathogenesis of VPA-induced hepatic steatosis in vivo and in vitro. Toxicology.

[47] Ye Q, Wang X, Wang Q, Xia M, Zhu Y, Lian F (2013). Cytochrome P4502E1 inhibitor, chlormethiazole, decreases lipopolysaccharide-induced inflammation in rat Kupffer cells with ethanol treatment. Hepatol Res..

[48] Bedada SK, Neerati P (2018). The effect of quercetin on the pharmacokinetics of chlorzoxazone, a CYP2E1 substrate, in healthy subjects. Eur J Clin Pharmacol.

[49] Jia P, Wang J, Wang L, Chen X, Chen Y, Li WZ (2013). TNF-alpha upregulates Fgl2 expression in rat myocardial ischemia/reperfusion injury. Microcirculation..

[50] Cheng SC, Huang WC, SP JH, Wu YH, Cheng CY (2019). Quercetin inhibits the production of IL-1beta-induced inflammatory cytokines and chemokines in ARPE-19 cells via the MAPK and NF-kappaB signaling pathways. Int J Mol Sci.

