# Supplementary material for: Stat4 rs7574865 polymorphism promotes the occurrence and progression of hepatocellular carcinoma via the Stat4/CYP2E1/FGL2 pathway
Source: Cell Death Dis. 2022 Feb 8;13(2):130. doi: 10.1038/s41419-022-04584-4 (PMC8826371; doi:10.1038/s41419-022-04584-4)
Supplement: Supplementary file 1 — Table S1 [file 41419_2022_4584_MOESM1_ESM.docx]

**Table S1 Logistic analysis of association of *stat4* rs7574865 genotypes/alleles with the risk of HCC in a Chinese population.**

| Group | Control N(%) | HCC N(%) | OR(95%CI) | *P* value |
| --- | --- | --- | --- | --- |
| Genotype  TT  TG  GG  Allele  Minor(T)  Major(G)  Recessive  TT+GT  GG  Dominant  TT  TG+GG | 45 (9.70)  205 (44.18)  214 (46.12)  295 (31.79)  633 (68.21)  250 (53.88)  214 (46.12)  45 (9.70)  419 (90.30) | 40 (8.02)  183 (36.67)  276 (55.31)  263 (26.35)  735 (73.65）  223 (44.69)  276 (55.31)  40 (8.02)  459 (91.98) | Reference  1.00(0.68-1.61)  1.45(0.91-2.30)  Reference  1.30 (1.07-1.59)  Reference  1.45 (1.12-1.86)  Reference  1.23 (0.79-1.93) | 0.541  **0.017**  **0.009**  **0.005**  0.366 |
| Total | 464 | 499 |  |  |

Logistic regression models were used to calculate the ORs, 95% CIs, and the corresponding *P* values of referent, allele, dominant, and recessive models controlling for age and sex as covariates. Significant associations are shown in bold. Control,Healthy person; HCC, hepatocellular carcinoma; OR, odd ratio; CI, confidence interval.
