# Supplementary material for: Stat4 rs7574865 polymorphism promotes the occurrence and progression of hepatocellular carcinoma via the Stat4/CYP2E1/FGL2 pathway
Source: Cell Death Dis. 2022 Feb 8;13(2):130. doi: 10.1038/s41419-022-04584-4 (PMC8826371; doi:10.1038/s41419-022-04584-4)
Supplement: Supplementary file 2 — Table S2 [file 41419_2022_4584_MOESM2_ESM.docx]

**Table S2 The basic characteristics and clinical parameters of studied subjects**

| **Characteristics** | **Control** | **HCC** |
| --- | --- | --- |
| Age(years) | 46.65±14.50 | 53.93±10.93 |
| Gender(M/F) | 359/141 | 388/112 |
| ALT(IU/L)(0-40) | 21.32±14.37 | 61.82±126.59 ^*^ |
| AST(IU/L)(0-40) | 20.69±12.53 | 83.73±203.65^*^ |
| ALB (g/L) (40-55) | 40.61±5.58 | 36.58±6.07^*^ |
| GGT(IU/L)(6-73) | 26.15±21.57 | 120.67±153.04^*^ |
| INR(0.8-1.2) | 0.91±0.12 | 1.05±0.36^*^ |
| TT (s) (11-22) | 14.50±2.01 | 15.62±2.28^*^ |
| APTT (s) (20-40) | 34.07±5.21 | 38.28±7.43^*^ |
| PT (s) (11-15) | 10.10±1.44 | 11.65±2.39^*^ |
| TBIL( μmol/L)(0-20) | 10.85±5.64 | 29.84±61.46^*^ |
| DBIL( μmol/L)(0-6) | 4.56±2.21 | 19.40±49.27 ^*^ |
| IBIL( μmol/L)(1.4-13.5) | 6.32±3.78 | 10.52±13.49^*^ |
| GLB (g/L) (25-35) | 24.91±4.45 | 28.15±6.20 ^*^ |
| TP (g/L) (65-85) | 65±8.55 | 64.62±7.34 |
| AFP (mg/L) (0-400) | NA | 1185.20±9776.70 |
| TBA(μmol/L)(0.1-15) | 5.15±6.19 | 25.99±36.45^*^ |

Abbreviation:**Control**, Healthy persons; **HCC**, hepatocellular carcinoma; **ALT**, alanine aminotransferase; **AST**, aspartate aminotransferase; **ALB**, albumin; **GGT**, gamma-glutamyl transferase; **INR,** [international standard ratio](http://abbr.dict.cn/International+standard+ratio/INR); **TT**, thrombin time; **APTT**, activated partial thromboplastin time; **PT**, prothrombin time; **TBIL**, total bilirubin; **DBIL,** direct bilirubin; **IBIL,** indirect bilirubin; **AFP,** alpha-foetoprotein. **GLB,** globulin; TP, total protein; **TBA,** total biliary acid. VS control,**P* < 0.05.
