# Supplementary material for: Stat4 rs7574865 polymorphism promotes the occurrence and progression of hepatocellular carcinoma via the Stat4/CYP2E1/FGL2 pathway
Source: Cell Death Dis. 2022 Feb 8;13(2):130. doi: 10.1038/s41419-022-04584-4 (PMC8826371; doi:10.1038/s41419-022-04584-4)
Supplement: Supplementary file 3 — Table S3 [file 41419_2022_4584_MOESM3_ESM.docx]

**Table S3 Hardy-Weinberg Equilibrium test for *stat4* rs7574865 in serum**

| Gene | SNP | Control(N=500) | | HCC(N=500) | |
| --- | --- | --- | --- | --- | --- |
|  |  | χ^2^ | *P* | χ^2^ | *P* |
| *stat4* | *rs*7574865 | 0.000491 | 0.982316 | 1.396544 | 0.237283 |
