# Supplementary material for: Stat4 rs7574865 polymorphism promotes the occurrence and progression of hepatocellular carcinoma via the Stat4/CYP2E1/FGL2 pathway
Source: Cell Death Dis. 2022 Feb 8;13(2):130. doi: 10.1038/s41419-022-04584-4 (PMC8826371; doi:10.1038/s41419-022-04584-4)
Supplement: Supplementary file 4 — Table S4 [file 41419_2022_4584_MOESM4_ESM.docx]

**Table S4 Prognostic factors for overall survival by univariate analyses in patients with HCC**

| **Variables** | **N (%)** | **HR** | **95% CI** | ***P value*** |
| --- | --- | --- | --- | --- |
| Age(year),>55/≤55 | 157(50.6)/155(49.4) | 0.981 | 0.692~1.391 | *0.9158* |
| Gender,Male/Female | 252(80.3)/62(19.7) | 0.862 | 0.567~1.325 | *0.5097* |
| Smoking,yes/no | 71(22.6)/243(77.4.1) | 1.173 | 0.776~1.799 | 0.4361 |
| Drinking,yes/no | 57(18.2)/257(81.8) | 1.276 | 0.820~2.072 | *0.2628* |
| ALT (U/L), >40/≤40 | 104(33.1)/210(66.9) | 2.168 | 1.650~3.601 | ***< 0.0001*** |
| AST (U/L), >40/≤40 | 135(43.0)/179(57.0) | 2.828 | 2.149~4.450 | ***< 0.0001*** |
| GGT (U/L), >73/≤73 | 142(45.2)/172(54.8) | 3.037 | 2.374~4.956 | ***< 0.0001*** |
| GLB (g/L), >35/≤35 | 38(12.1)/276(87.9) | 1.555 | 1.018~2.919 | ***0.0479*** |
| PT (s), >15/≤15 | 45(14.3)/269(85.7) | 2.838 | 2.325~13.20 | ***0.0001*** |
| APTT (s), >40/≤40 | 122(38.8)/192(61.2) | 1.167 | 0.798~1.730 | *0.4177* |
| D-D (μg/L), >1/≤1 | 115(36.6)/199(63.4) | 2.389 | 1.903~4.695 | ***< 0.0001*** |
| FIB (g/L), >4/≤4 | 22(7.2)/282(92.8) | 2.383 | 1.673~7.876 | ***0.0011*** |
| DBIL (μmol/L), >6/≤6 | 170(59.9.1)/114(41.9) | 2.211 | 1.440~3.002 | ***0.0001*** |
| INR , >1.2/≤1.2 | 35(11.8)/263(88.2) | 2.348 | 1.804~6.233 | ***0.0001*** |
| TBA(μmol/L) , >15/≤15 | 95(44.8)/117(55.2) | 1.027 | 0.681~1.551 | *0.8972* |
| IBIL (μmol/L), >13.5/≤13.5 | 57(21.2)/225(79.8) | 1.277 | 0.820~2.068 | *0.2649* |
| CA199(>25/≤25) | 79(39.9)/119(60.1) | 2.445 | 1.662~4.217 | ***< 0.0001*** |
| CA125(>35/≤35) | 59(33.2)/119(66.8) | 2.723 | 1.964~5.566 | ***< 0.0001*** |
| AFP (mg/L), ≥400/<400 | 86(29.3)/208(70.7) | 3.147 | 2.817~6.505 | ***< 0.0001*** |

Abbreviation:**ALT**, alanine aminotransferase; **AST**, aspartate aminotransferase; **GGT**, gamma- glutamyl transferase; **GLB**, globulin; **PT**, prothrombin time; **APTT**, activated partial thromboplastin time; **TT**, thrombin time; **INR,** [international standard ratio](http://abbr.dict.cn/International+standard+ratio/INR); **D-D**, *D*-dimer; **FIB**, fibrinogen; **TBA,** total biliary acid; **DBIL,** direct bilirubin; **IBIL,** indirect bilirubin; **AFP,** alpha-foetoprotein. *P*-value was calculated using univariate analyses with a log-rank test. *P* < 0.05 was considered statistically significant (two-tailed test).
