# Supplementary material for: Stat4 rs7574865 polymorphism promotes the occurrence and progression of hepatocellular carcinoma via the Stat4/CYP2E1/FGL2 pathway
Source: Cell Death Dis. 2022 Feb 8;13(2):130. doi: 10.1038/s41419-022-04584-4 (PMC8826371; doi:10.1038/s41419-022-04584-4)
Supplement: Supplementary file 5 — Table S5 [file 41419_2022_4584_MOESM5_ESM.docx]

**Table S5 Prognostic factors for survival time by multivariate analysis in HCC patients**

| Variable | b | SE | Wald^2^ | *P* | HR | 95% CI |
| --- | --- | --- | --- | --- | --- | --- |
| PT | 0.915 | 0.301 | 9.240 | 0.002 | 2.496 | 1.384-4.501 |
| D-D | 0.646 | 0.203 | 10.137 | 0.001 | 1.908 | 1.282-2.839 |
| AST | 0.845 | 0.201 | 17.624 | <0.0001 | 2.328 | 1.569-3.454 |
| GGT | 0.580 | 0.204 | 8.112 | 0.004 | 1.786 | 1.198-2.661 |
| FIB | 0.961 | 0.307 | 9.766 | 0.002 | 2.613 | 1.431-4.773 |
| AFP | 0.776 | 0.187 | 17.129 | <0.0001 | 2.172 | 1.504-3.136 |
| rs7574865GG | 2.101 | 0.721 | 8.497 | 0.004 | 8.177 | 1.991-33.590 |

Abbreviation: **PT**, prothrombin time; **D-D**, D-dimer; **AST**, aspartate aminotransferase; **GGT**, gamma-glutamyl transferase; **FIB**, fibrinogen; **AFP**, alpha-foetoprotein. *P*-value was calculated by multivariate analysis using Cox hazard regression model. *P* < 0.05 was considered statistically significant.
