# Supplementary material for: Stat4 rs7574865 polymorphism promotes the occurrence and progression of hepatocellular carcinoma via the Stat4/CYP2E1/FGL2 pathway
Source: Cell Death Dis. 2022 Feb 8;13(2):130. doi: 10.1038/s41419-022-04584-4 (PMC8826371; doi:10.1038/s41419-022-04584-4)
Supplement: Supplementary file 7 — Figure legends [file 41419_2022_4584_MOESM7_ESM.docx]

**Fig.1 Association of the *stat4* rs7574865 genetic polymorphism with the risk of HCC. A-B** The relationship between the *stat4* genetic polymorphism and clinical parameters: GGT (Control: *TT*+*TG* N*=*127, *GG* N=159; HCC: *TT*+*TG* N*=*230, *GG* N*=*269) and AST (Control: *TT*+*TG* N*=*127, *GG* N*=*159; HCC: *TT*+*TG* N=230, 269 *GG* N*=*269) (^*^*P* < 0.05*, P value* was calculated by Mann–Whitney U test). **C** Kaplan-Meier survival curves for patients with HCC by *stat4* genetic polymorphism (Serum: *TT*+*TG* N*=*136, *GG* N*=*178) *P* values are depicted by Cox-Mantel log-rank test. **D** STAT4 expression in HCC patients and controls in human sera (Control:N=60; HCC:N=65); **E-F** STAT4 expression with different genotypes in human serum (HCC: *TT*+*TG* N*=*42, *GG* N*=*23 and Control:*TT*+*TG* N*=*38, *GG* N*=*22);**G** STAT4 expression in HCC patients and controls in human liver tissue (Control: N=56; HCC:N= 42); **H-I** STAT4 expression with different genotypes in human liver tissue (HCC: *TT*+*TG* N*=*13, *GG* N*=*31; Control: *TT*+*TG* N*=*25, *GG* N*=*31) ^*^*P* < 0.05, ^**^*P* < 0.01, ^***^*P* < 0.001 was assessed by Student’s t test. **J, L** Survival curves of HCC patients with high and low STAT4 expression (Serum: N=65; Liver tissue: N=42); **K** Kaplan-Meier survival curves for patients with HCC by *stat4* genetic polymorphism (liver tissue: *TT*+*TG* N*=*13, *GG* N*=*31). *P* values are depicted by Cox-Mantel log-rank test.

**Fig.2 The effect of STAT4 on apoptosis, growth cycle, migration and invasion in HepG2 and L02 cells.** **A** The transfection efficiency was determined under a fluorescent microscope 48 h after transfection. The fluorescent siRNA (green) is evident in HepG2 and L02 cells. **B** Inhibition of cell proliferation by MTT assay at different time in HepG2 and L02 cells. **C** mRNA expression of STAT4 in HepG2 and L02 cells estimated by qPCR. **D** Expression of STAT4 and GAPDH in protein extracts from cultured HepG2 and L02 cells after 48 h by Western blot. Quantification of STAT4 is shown in the right picture. Data are representative of three independent experiments. **E** STAT4 siRNA inhibited the migration and invasion of HepG2 and L02 cells lines. **F** STAT4 siRNA promoted apoptosis in HepG2 and L02 cells. **G** STAT4 siRNA affected the growth cycle in HepG2 and L02 cells. Scale bar:50 µm, 100 µm, N=3.*^*^P<*0.05*,^**^P<*0.01*, ^***^P<*0.001 was assessed by one-way ANOVA*.*

**Fig.3 Schematic diagram to identify differentially expressed proteins resulting from the STAT4 polymorphism: possible regulation of the CYP2E1/FGL2 pathway.** **A** An enrichment analysis of the KEGG pathway for differentially expressed proteins in hepatofibrotic tissue of HCC patients. **B** Venn diagram showing the number of differential proteins in hepatofibrotic tissue of HCC patients. **C** Functional classification of differentially expressed proteins affected by the STAT4 polymorphism and HCC: inflammation, angiogenesis, proliferation, immunity and metabolism. **Inflammation:** **MVP**, Major vault protein, **STAT1**, Signal transducer and activator of transcription 1. **Angiogenesis: PFN**, Profilin-1; **MME**, Neprilysin; **NPNT**, Nephronectin. **Proliferation: INHBE**, Inhibin beta E chain; **BBOX1**, Gamma-buty robetaine dioxyge1. **Immunity:** **LGALS9**, Galectin-9; **FGL2**, Fibroleukin (Fibrinogen-like protein 2); **TAPBP**, Tapasin (TAP-binding protein); **PARP4**-Poly [ADP-ribose] polymerase 4; **ELMO2**, Engulfment and cell motility protein 2. **Metabolism:** **BCAT2**, Branched chain amino acid aminotransferase; **DHRS2**, Dehydrogenase/reductase SDR family member 2; **GYS2**, Glycogen synthase; **G6PD**, Glucose-6-phosphate 1-dehydrogenase;**RBP4**, Retinol binding protein 4; **BCHE**, Choline-esterase. **D** Kaplan-Meier curve of overall survival for patients grouped by median of FGL2 expression levels, *P* values are depicted by Cox-Mantel log-rank test. **E-F** The correlation between the expression of FGL2 and the activity of CYP2E1 (V_max_ and Cl_int_) in human HCC liver tissue, R and *P* values by Pearson’s correlation test are depicted. **G** The expression of FGL2 on the median of V_max_ and Cl_int_ as the cutoff point, in HCC liver tissue by Western blot. GAPDH in each lane served as an internal control for normalization (N=3),*^*^P <* 0.05 was assessed by Student’s t test*.*

**Fig.4 The regulation by STAT4 of CYP2E1. A-D** The correlation between content of STAT4 and activity of CYP2E1 (V_max_ and Cl_int_) was analyzed. The result revealed that content of STAT4 was significantly positively correlated with the V_max_ and Cl_int_ in control and HCC liver tissues (N=42, R and *P* values by Pearson’s correlation test are depicted). **E-F** The expression of STAT4 and CYP2E1 in HepG2 and L02 cells in the IL-12 treated group and the STAT4 siRNA group by western blot (N=3). GAPDH in each lane served as an internal control for normalization. HepG2 and L02 cells for 48 h with blank control group, negative control group, IL-12 group and siRNA-STAT4 group. Data are representative of three independent experiments. **G The** effect of STAT4 on CYP2E1 promoter activity was detected by double luciferase reporter gene.Promoter-NC+TFs: STAT4 (TFs) and CYP2E1 promoter negative control plasmid; (CYP2E1 promoter+TFs-NC group): CYP2E1 promoter and STAT4-negative control plasmid (TFs-NC) group; CYP2E1 promoter-NC and TFs-NC plasmid group; CYP2E1 promoter and TFs (STAT4); Promoter: CYP2E1 promoter normal plasmid group (N=3).*^*^P <* 0.05*, ^**^P <* 0.01*,^***^P <* 0.001 was assessed by one-way ANOVA.

**Fig. 5 The relationship between FGL2 and HCC. A** Comparison of FGL2 expression levels between the normal group (N=34) and the HCC group (N=42); **B** Comparison of FGL2 expression levels between the low STAT4 group (N=21) and the high STAT4 group (N=21); **C** The expression of FGL2 in liver tissue by western blot (N=6), *^*^P <* 0.05*, ^***^P <* 0.001 was assessed by Student’s t test. **D** ROC curve analysis for FGL2, AUC indicated area under ROC curve,. **E-I** The relationship between the expression of FGL2 and clinical indicators (AFP, ALT, AST, GLB, GGT). *^*^P <* 0.05*,^**^P <* 0.01*,^***^P <* 0.001 was assessed by Student’s t test.

**Fig. 6 CYP2E1 regulates STAT4-mediated FGL2 expression. A** Experimental scheme of the effect of CMZ on FGL2 in BALB/c mice; **B** Representative images of macroscopic liver (top) and H&E staining (bottom) (scale bar, 50 µm); **C** The tumor weight ;sham group (N=10), model group (N=12), CMZ group (N=12), *^*^P <* 0.05*,^**^P <* 0.01 was assessed by one-way ANOVA. **D** The expression of FGL2 in liver tissues in an orthotopic transplantation tumor model of H22 cells in BALB/c mice by western blot (N=3). **E** The 6-hydroxy-CZX formation (N=6), *^*^P <* 0.05*,^**^P <* 0.01 was assessed by one-way ANOVA. **F** The effect of *cyp2e1* knockout in mice was identified by PCR. Mice no 1, no 2, no 3 and no 5 only showed bands around 520 bp, and no bands around 464 bp, which were mutant homozygous (N=6). Mice no. 4 and no. 6 exhibited two bands near 464 bp and 520 bp, respectively, indicating that they were heterozygous. **G** The expression of FGL2 in liver tissues in (N=3). *^**^P <* 0.01 was assessed by Student’s t test .

**Fig.7 Graphical representation of the possible mechanism by which the *stat4* genetic polymorphism affects HCC.** STAT4 has dual roles during HCC (i) STAT4 directly promotes HepG2 cell proliferation, migration and invasion, and affects apoptosis and cell cycle; and (ii) Polymorphism rs7574865 participates the occurrence and progression of HCC by regulating the CYP2E1/FGL2 pathway.
