# Supplementary material for: Stat4 rs7574865 polymorphism promotes the occurrence and progression of hepatocellular carcinoma via the Stat4/CYP2E1/FGL2 pathway
Source: Cell Death Dis. 2022 Feb 8;13(2):130. doi: 10.1038/s41419-022-04584-4 (PMC8826371; doi:10.1038/s41419-022-04584-4)
Supplement: Supplementary file 8 — Supplementary Table legends. [file 41419_2022_4584_MOESM8_ESM.docx]

**Table S1 Logistic analysis of association of *stat4* rs7574865 genotypes/alleles with the risk of HCC in a Chinese population.**

Logistic regression models were used to calculate the ORs, 95% CIs, and the corresponding *P* values of referent, allele, dominant, and recessive models controlling for age and sex as covariates. Significant associations are shown in bold. Control,Healthy person; HCC, hepatocellular carcinoma; OR, odd ratio; CI, confidence interval.

**Table S2 The basic characteristics and clinical parameters of studied subjects**

Abbreviation:**Control**, Healthy persons; **HCC**, hepatocellular carcinoma; **ALT**, alanine aminotransferase; **AST**, aspartate aminotransferase; **ALB**, albumin; **GGT**, gamma-glutamyl transferase; **INR,** [international standard ratio](http://abbr.dict.cn/International+standard+ratio/INR); **TT**, thrombin time; **APTT**, activated partial thromboplastin time; **PT**, prothrombin time; **TBIL**, total bilirubin; **DBIL,** direct bilirubin; **IBIL,** indirect bilirubin; **AFP,** alpha-foetoprotein. **GLB,** globulin; TP, total protein; **TBA,** total biliary acid. VS control,**P* < 0.05.

**Table S3 Hardy-Weinberg Equilibrium test for *stat4* rs7574865 in serum**

**Table S4 Prognostic factors for overall survival by univariate analyses in patients with HCC**

Abbreviation:**ALT**, alanine aminotransferase; **AST**, aspartate aminotransferase; **GGT**, gamma- glutamyl transferase; **GLB**, globulin; **PT**, prothrombin time; **APTT**, activated partial thromboplastin time; **TT**, thrombin time; **INR,** [international standard ratio](http://abbr.dict.cn/International+standard+ratio/INR); **D-D**, *D*-dimer; **FIB**, fibrinogen; **TBA,** total biliary acid; **DBIL,** direct bilirubin; **IBIL,** indirect bilirubin; **AFP,** alpha-foetoprotein. *P*-value was calculated using univariate analyses with a log-rank test. *P* < 0.05 was considered statistically significant (two-tailed test).

**Table S5 Prognostic factors for survival time by multivariate analysis in HCC patients**

Abbreviation: **PT**, prothrombin time; **D-D**, D-dimer; **AST**, aspartate aminotransferase; **GGT**, gamma-glutamyl transferase; **FIB**, fibrinogen; **AFP**, alpha-foetoprotein. *P*-value was calculated by multivariate analysis using Cox hazard regression model. *P* < 0.05 was considered statistically significant.
