# Supplementary material for: Stat4 rs7574865 polymorphism promotes the occurrence and progression of hepatocellular carcinoma via the Stat4/CYP2E1/FGL2 pathway
Source: Cell Death Dis. 2022 Feb 8;13(2):130. doi: 10.1038/s41419-022-04584-4 (PMC8826371; doi:10.1038/s41419-022-04584-4)
Supplement: Supplementary file 9 — Highlights [file 41419_2022_4584_MOESM9_ESM.doc]

**Highlights**

1. STAT4 genetic polymorphism may cause STAT4 protein content change and affect the occurrence and progression of HCC.

2. STAT4 possible participates in the occurrence and progression of HCC by regulating CYP2E1.

3. CYP2E1 regulates the expression of FGL2 in HCC patients. With the high expression of FGL2 in liver fibrosis tissue, having a poor prognosis and an increased risk of death.

4. STAT4 genetic polymorphism is involved in the occurrence andprogression of HCC, and its mechanism may be related to STAT4's regulationon CYP2E1 and further affect the expression of FGL2.
